# Natural history and mid-term prognosis of severe tricuspid regurgitation: A cohort study

**DOI:** 10.3389/fcvm.2022.1026230

**Published:** 2023-01-09

**Authors:** Daryoush Samim, Fabien Praz, Baptiste Cochard, Nicolas Brugger, Andrea Ruberti, Joanna Bartkowiak, Noé Corpataux, David Reineke, Thomas Pilgrim, Stephan Windecker, Peter Martin Wenaweser, Mirjam G. Wild

**Affiliations:** ^1^Department of Cardiology, Bern University Hospital, Bern, Switzerland; ^2^Department of Cardiac Surgery, Bern University Hospital, Bern, Switzerland; ^3^HerzKlinik Hirslanden, Zürich, Switzerland; ^4^Medizinische Klinik und Poliklinik I, LMU University Hospital, Munich, Germany

**Keywords:** valvular heart disease, tricuspid regurgitation, lead-induced TR, TTVI, echocardiography

## Abstract

**Objectives:**

The objective of this study was to characterize a population of patients with severe tricuspid regurgitation (TR) evaluated at a tertiary care center, assess mid-term clinical outcomes, and identify prognostic factors.

**Background:**

The impact of TR on morbidity and mortality is increasingly recognized. Clinical characteristics and long-term outcomes of patients suffering from TR remain unclear.

**Methods:**

This is a retrospective observational single-center study from a tertiary care hospital including patients with echocardiographic diagnosis of severe TR between January 2017 and December 2018. We used the Kaplan–Meier method to estimate survival for up to 4 years. After excluding patients with tricuspid valve (TV) intervention and surgery during follow-up, a multivariate analysis was performed to assess predictors of 2-year mortality using the Cox regression model.

**Results:**

A total of 278 patients (mean age 74.9 ± 13.7 years, 47.8% female) with severe TR were included in the study. The majority (83.1%; *n* = 231) had secondary TR. Comorbidities such as atrial fibrillation (AFib) (68.0%; *n* = 189), severe renal failure (44.2%; *n* = 123), pulmonary hypertension (PHT) (80.9%; *n* = 225), and right ventricular (RV) dysfunction (59.7%; *n* = 166) were highly prevalent. More than half of patients with a cardiac implantable electronic device (CIED) (54.3%; *n* = 44) showed echocardiographic signs of lead-leaflet interaction causing or contributing to TR. The estimated 2- and 4-year all-cause mortality was 50 and 69%, respectively. Using multivariate analysis, age, severe renal failure, heart failure with reduced ejection fraction (HFrEF), and vena contracta width ≥14 mm were identified as predictors of 2-year mortality. Nine percent (*n* = 25) of the study cohort underwent transcatheter or surgical treatment for TR during follow-up.

**Conclusion:**

Our study shows the high burden of morbidity and the dismal survival of patients with severe TR. It also highlights the extent of the therapeutic need, since the vast majority of patients were left untreated. Additionally, CIED RV lead-associated TR was prevalent suggesting a need for more attention in clinical routine and research.

## Introduction

Tricuspid regurgitation (TR) is one of the most common valvular heart diseases. TR of any severity affects more than 65% of the general population worldwide and the prevalence of clinically significant (moderate or higher grade) TR is comparable to the one of aortic stenosis in patients >75 years of age (1, 2). However, due to challenges in imaging, lack of minimal-invasive treatment strategies, and misconception of its clinical course, TR has long been considered less clinically relevant than left-sided heart valve disease. Over the last few years, growing evidence on TR long-term negative impact on morbidity and mortality (1, 3, 4), as well as the emergence of new treatment options have resulted in an increasing interest. Nevertheless, the medical literature regarding patients with severe TR outside of interventional studies is scarce and little is known about the clinical characteristics and natural history of this patient population.

This study aimed to characterize patients with severe TR referred to a tertiary care center, assess mid-term clinical outcomes, and identify prognostic factors.

## Materials and methods

### Study population

This was a retrospective single-center cohort study conducted at a tertiary care hospital. All echocardiographic reports [transthoracic echocardiography (TTE) and transesophageal echocardiography (TEE)] between January 2017 and December 2018 were automatically screened to identify consecutive patients with severe TR. Echocardiographic images were evaluated by experienced echocardiographers and severe TR was independently confirmed by a second physician. Data were then cleaned for duplicates and falsely identified patients. All parameters were collected from hospital electronic files.

### Definitions

Severe TR was defined in an integrative way as a vena contracta width ≥7 mm, hepatic systolic backflow, EROA ≥40 mm^2^, and regurgitant volume ≥45 mL according to the American society of echocardiography (5) and the 2017 ESC/EACTS Guidelines for the management of valvular heart disease (6). Severe TR was further subclassified according to vena contracta width into massive and torrential TR using the previously described five-grade scheme (7). RV dysfunction was defined as TAPSE < 17 mm or S-DT I < 9.5 cm/s and pulmonary hypertension (PHT) as an estimated pulmonary artery systolic pressure (PASP) >40 mmHg based on RV/RA-gradient and central venous pressure. RV-PA coupling ratio was calculated as TAPSE/PASP (8, 9). RV dilatation was defined as RV end-diastolic base diameter >41 mm. MR was graded using a three-scale scheme (mild/moderate/severe) according to the EACVI Guidelines (10–12). Severe renal failure was defined as GFR < 30 mL/min/1.73 m^2^ on a laboratory value taken on the day of echocardiographic diagnosis of severe TR. Chronic pneumopathy was defined as COPD or interstitial lung disease.

### Clinical outcomes

Two- and four-year mortality was assessed using the patient’s medical records, as well as the national registry of deaths. For all patients alive, the date of last contact was considered.

### Statistical analysis

Statistical analyses were performed using SPSS version 25.0 for Windows. Results were expressed as absolute number and/or percentage for categorical variables and mean (± SD) or median (interquartile range) for continuous variables. Comparisons were performed using the chi-square test or Fisher’s exact test for categorical variables and Student’s *t*-test or Kruskal–Wallis test for continuous variables. A multivariate analysis was performed to assess predictors of 2-year mortality using the Cox regression model and the results were expressed as hazard ratio (HR) and 95% confidence interval (CI). Variables with *p* < 0.05 and variables associated with mortality in the literature were included in the multivariate model after the exclusion of collinearity. Patients with tricuspid valve (TV) intervention or surgery during follow-up were excluded from the multivariate analysis.

We used the Kaplan–Meier method and log-rank test to estimate survival for up to 4 years. Patients were divided into three groups according to their treatment during follow-up: (1) medical treatment only, (2) TV intervention, and (3) TV surgery.

### Ethical statement

The local Ethics Committee (Kantonale Ethikkommission Bern) approved this study (TricObs Registry; KEK-Nr. 2019-02147). The study was performed in agreement with the Helsinki declaration and in accordance with the applicable Swiss legislation. Ethics Committee provided a waiver of consent. All patients having refused the use of their medical data were excluded.

## Results

**FIGURE 1:**
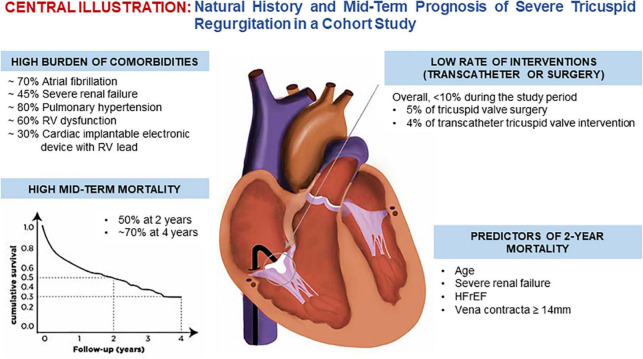
Central illustration.

Overall, 25,295 echocardiography reports generated between January 2017 and December 2018 were screened for diagnosis of severe TR. After cleaning for duplicates and falsely identified patients, the proportion of moderate or severe TR among patients ≥70 was 4.3%, while 2.5% had severe TR. A total of 278 patients with severe TR were included into the study (Supplementary Figure 1). Figure 1 is the central illustration and summarizes the main findings of the study.

### Clinical characteristics

The baseline clinical characteristics of the cohort are summarized in Table 1. Mean age was 74.9 ± 13.7 years and 47.8% were female. The prevalence of co-morbidities was high: severe renal failure was common (44.2%; *n* = 123) and 18.7%, (*n* = 52) had a chronic pneumopathy. Atrial fibrillation (AFib) was found in more than two thirds of the patients (68.0%, *n* = 189) and coronary artery disease (CAD) in 42.8% (*n* = 119). One-third (32.7%; *n* = 91) had undergone open-heart surgery and among them 7.2% (*n* = 20) had combined procedures (Supplementary Table 1). The two most commonly performed surgical procedures were aortic valve replacement (*n* = 40) and coronary artery bypass grafting (*n* = 35). Recurrent severe TR (*n* = 3) after TV repair was rare and no patient had undergone TV replacement.

**TABLE 1:** Baseline clinical characteristics.

N	278
Age (year)	74.9 ± 13.7
BMI (kg/m^2^)	25.4 ± 5.0
Female	133 (47.8)
CAD	119 (42.8)
Previous open-heart surgery	91 (32.7)
Previous tricuspid valve surgery (repair)	5 (1.8)
Previous TAVR	25 (9.0)
Previous mitral TEER	25 (9.0)
Previous TTVr	10 (3.6)
Severe renal failure (GFR < 30 mL/min/1.73 m^2^)	123 (44.2)
Creatinine (umol/l)	107 (85–155)
NT-proBNP (pg/mL)	4,580 (2,203–4,580)
Chronic pneumopathy	52 (18.7)
Malignancy	31 (11.2)
Atrial fibrillation	189 (68.0)
ACHD	10 (3.6)
Acute TR	13 (4.7)
OHT/VAD	14 (5.0)
RV Lead	81 (29.1)

Results are expressed as percentage for categorical variables and mean (± SD) or median. (IQR) for continuous variables or number of patients (percentage) for categorical data. CAD, coronary artery disease; TAVR, transcatheter aortic valve replacement; mitral TEER, mitral transcatheter edge-to-edge repair; TTVr, transcatheter tricuspid valve repair; ACHD, adult congenital heart disease; OHT, orthotopic heart transplantation; VAD, ventricular assist device; Chronic pneumopathy, COPD or interstitial lung disease.

**FIGURE 2:**
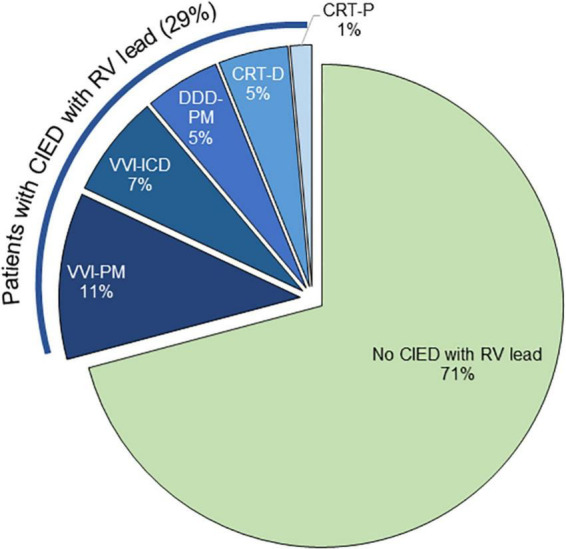
Distribution of cardiac implantable electronic device (CIED) in the cohort.

Twenty-five patients each had undergone previous transcatheter aortic valve replacement (TAVR) (9.0%) and mitral transcatheter edge-to-edge repair (TEER) (9.0%); 10 patients had a history of previous transcatheter TV intervention (3.6%). Adults with congenital heart disease (ACHD) constituted a small portion of the overall cohort (3.6%; *n* = 10). In 13 patients (4.7%), TR was attributable to an acute event such as pulmonary embolism, while almost the same proportion of patients (5.0%; *n* = 14) was either recipient of orthotopic heart transplantation (OHT) or carrier of a ventricular assistance device (VAD). Almost one-third (29.1%; *n* = 81) of the patients had a cardiac implantable electronic device (CIED) RV lead (Figure 2). The most common were single chamber (RV lead) pacemakers (11.2%; *n* = 31) and implantable cardioverter defibrillators (ICD) (6.8%; *n* = 19), followed by dual chamber pacemakers (5.0%; *n* = 14) and defibrillators with cardiac resynchronization therapy (CRT-D) (4.7%; *n* = 13). Cardiac resynchronization therapy pacemakers (CRT-P) were uncommon (1.4%; *n* = 4).

### Echocardiographic characteristics

Baseline echocardiographic data of the cohort are summarized in Table 2. The vast majority had severe TR (92.8%), while massive (5.8%) or torrential TR (1.4%) were less frequent in our cohort. HFrEF (LVEF ≤ 40%) was diagnosed in 27.0% (*n* = 75) of patients and 59.7% (*n* = 166) had RV dysfunction. The majority of patients showed signs of PHT (80.9%; *n* = 225) and the RV was dilated in 78.1% (*n* = 217). Every third patient (34.2%; *n* = 95) had moderate MR, while 9.7% (*n* = 27) had severe MR. TR was of secondary etiology in the majority of the patients (83.1%; *n* = 231), mixed in 11.5% (*n* = 32) and primary in 3.6% (*n* = 10). For a few patients (1.8%; *n* = 5) no exact mechanism causing TR could be identified on TTE. Among 81 patients (29.1%) with a CIED RV lead, more than half (53.6%) had signs of lead-leaflet interaction that was suspected of causing or contributing to TR.

**TABLE 2:** Baseline echocardiographic data.

N	278
LVEF (%)	49.7 ± 15.3
RV/RA-gradient (mmHg)	42.2 ± 18.1
TV annulus (cm)	4.4 ± 0.7
Secondary TR	231 (83.1)
**TR etiology**	
Primary	10 (3.6)
Secondary	231 (83.1)
Mixed	32 (11.5)
Unclear	5 (1.8)
TAPSE (mm)	15.3 ± 6.3
RV S-TDI (cm/s)	9.4 ± 3.4
Vena contracta width (mm)	9.0 ± 3.2
Severe TR (vena contracta width 7–13 mm)	258 (92.8)
Massive TR (vena contracta width 14–20 mm)	16 (5.8)
Torrential TR (vena contracta width ≥21 mm)	4 (1.4)
EROA (mm^2^)	46.1 ± 24.3
Regurgitant volume of TR (mL)	38.2 ± 14.5
IVC diameter (mm)	23.3 ± 6.1
LV systolic dysfunction (LVEF < 50%)	98 (35.3)
HFrEF (LVEF ≤ 40%)	75 (27.0)
PASP (mmHg)	55 ± 19
PHT (estimated PASP > 40 mmHg)	223 (80.2)
TAPSE/PASP (mm/mmHg)	0.30 ± 0.18
RV dysfunction (TAPSE < 17 mm or RV S-TDI < 9.5 cm/s)	166 (59.7)
RV dilatation (RVEDd base > 41 mm)	217 (78.1)
Hepatic systolic backflow	176 (63.3)
CIED RV lead	81 (29.1)
**Mitral regurgitation**	
None	18 (6.5)
Mild	138 (49.6)
Moderate	95 (34.2)
Severe	27 (9.7)

Results are expressed as mean ± SD or median (IQR) for continuous variables for continuous variables or number of patients (percentage) for categorical data. LVEF, left ventricular ejection fraction; TAPSE, tricuspid annular plane excursion; S-TDI, RV systolic-tissue doppler imaging; EROA, effective regurgitant orifice area; IVC, inferior vena cava inferior; HFrEF, heart failure with reduced ejection fraction; PHT, pulmonary hypertension; CIED, cardiac implantable electronic device.

### Mid-term survival

**FIGURE 3:**
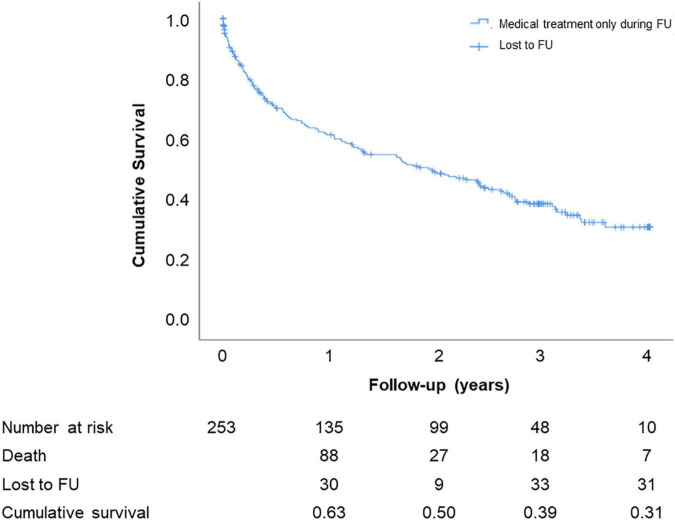
Estimated cumulative survival (Kaplan–Meier curve) up to 4-year follow-up.

The mean follow-up period was 1.5 ± 1.4 years. Two-year follow-up was available in 231 patients (83.1%), 3-year follow-up in 192 patients (69.1%), and 4-year follow-up in 151 (54.3%). Kaplan–Meier analysis showed cumulative mortality of 50% and almost 70% at 2 and 4 years, respectively, in conservatively treated patients (Figure 3). Mean survival was 2.1 ± 0.1 years.

### Predictors of 2-year mortality

**FIGURE 4:**
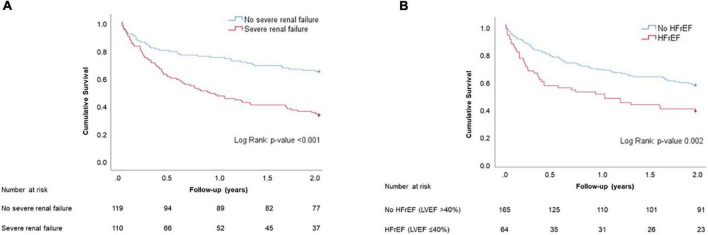
Panel **(A)**: Estimated cumulative survival (Kaplan–Meier curves) of patients with and without severe renal failure up to 2-year follow-up. Panel **(B)**: Estimated cumulative survival (Kaplan–Meier curves) of patients with and without HFrEF up to 2-year follow-up.

Patients who had undergone TV intervention or surgery during the follow-up period were excluded from this analysis. Compared with survivors, deceased patients at 2 years were older, had a higher incidence of severe renal failure, chronic pneumopathy, HFrEF, and PHT, had a wider vena contracta, had a lower RV-PA coupling ratio (TAPSE/PASP), were more likely to have ACHD and had higher serum creatinine and NT-proBNP levels (Table 3). Using multivariate analysis age (HR 1.05; 95% CI 1.02–1.07), severe renal failure (HR 2.21; CI 1.22–4.03), HFrEF (HR 1.97; CI 1.06–3.66), and TR vena contracta width ≥14 mm (HR 3.47; CI 1.19–10.12) predicted 2-year mortality (Table 4). The corresponding Kaplan–Meier curves are shown in Figures 4A, B. Patients with and without CIED RV lead had similar 2-year survival (50.6 vs. 50.7%) (Supplementary Figure 2).

**TABLE 3:** Characteristics of surviving and deceased patients at 2 years.

	Alive (*n* = 102)	Deceased (*n* = 115)	*P*-value
Age (years)	70.2 ± 15.5	77.6 ± 10.1	**<0.001**
Sex (Female)	44.1	50.4	0.352
Obesity (BMI ≥ 30 kg/m^2^)	25.7	29.2	0.572
ACHD	6.9	0.9	**0.019**
OHT/VAD	9.8	3.5	0.058
Acute TR	4.9	4.3	0.846
Secondary TR	85.1	90.7	0.213
CAD	36.3	46.1	0.143
Previous open-heart surgery	36.3	26.1	0.105
Previous tricuspid valve surgery	2.0	0.9	0.602
Previous TAVR	8.8	9.6	0.850
Previous mitral TEER	6.9	8.7	0.616
Previous TTVr	2.0	2.6	0.751
Severe renal failure (GFR < 30 mL/min/1.73 m^2^)	31.4	61.7	**<0.001**
Creatinine (umol/l)	105 (80–131)	126 (91–191)	**0.006**
Chronic pneumopathy	12.7	25.2	**0.020**
Malignancy	8.8	15.7	0.128
Atrial fibrillation	67.3	70.4	0.622
CIED RV lead	31.4	33.0	0.793
Severe MR	5.9	13.0	0.075
HFrEF (LVEF ≤ 40%)	19.6	34.8	**0.013**
NT-proBNP (pg/mL)	2,285 (1,024–5,211)	6,683 (4,417–10,209)	**<0.001**
PHT	71.1	87.2	**0.003**
TAPSE/PASP (mm/mmHg)	0.35 (0.22)	0.25 (0.13)	**0.001**
TAPSE/PASP ≤ 0.31	53.9	74.7	**0.003**
RV dilatation	81.4	79.1	0.679
RV dysfunction	68.2	77.0	0.175
Vena contracta width (mm)	8 (7–10)	9 (7–11)	**0.036**
Vena contracta width ≥14 mm	5.1	9.6	0.211
EROA (mm^2^)	49 (31–62)	42 (33–52)	0.641
Regurgitant Volume of TR (mL)	43 (17)	35 (10)	0.309
VCI diameter (mm)	23 (6)	24 (6)	0.073
Backflow in liver veins	64.7	70.4	0.368

Results are expressed as percentage for categorical variables and mean (± SD) or median (IQR) for continuous variables. Between-group comparisons using chi-square test or Fisher’s exact test for categorical variables and Student’s *t*-test or Kruskal–Wallis test for continuous variables. NB: Lost to follow-up at 2 years: *n* = 39. Patients with TV intervention or surgery during 2-year follow-up were excluded: *n* = 22. CAD, coronary artery disease; TAVR, transcatheter aortic valve replacement; mitral TEER, mitral transcatheter edge-to-edge repair; TTV, transcatheter tricuspid valve repair; ACHD, adult congenital heart disease; OHT, orthotopic heart transplantation; VAD, ventricular assist device. Chronic pneumopathy, COPD or interstitial lung disease.

**TABLE 4:** Predictors of 2-year mortality: Multivariate analysis.

	Mortality HR (95% CI)	*P*-value
Age	1.05 (1.02–1.08)	**0.001**
ACHD	1.48 (0.16–13.42)	0.726
Severe renal failure (GFR < 30 mL/min/1.73 m^2^)	2.21 (1.22–4.03)	**0.009**
Chronic pneumopathy	1.18 (0.63–2.24)	0.606
PHT	1.67 (0.57–4.89)	0.348
TAPSE/PASP ≤ 0.31	0.953 (0.51–1.80)	0.881
HFrEF (LVEF ≤ 40%)	1.973 (1.06–3.66)	**0.031**
Vena contracta width ≥14 mm	3.47 (1.19–10.12)	**0.023**

Cox regression (proportional hazards regression) for mortality analysis at 2 years. ACHD, adult congenital heart disease; PHT, pulmonary hypertension; PASP, estimated pulmonary artery systolic pressure; Chronic pneumopathy: COPD or interstitial lung disease. NB: Lost to follow-up at 2 years: *n* = 39. Patients with TV intervention or surgery during 2-year follow-up were excluded: *n* = 22.

### Tricuspid valve intervention or surgery during follow-up

Fourteen (5.0%) and 11 patients (4.0%) underwent either TV surgery or intervention during follow-up, respectively. The type of surgery or intervention performed during the 4-year follow-up period is shown in Supplementary Table 2. Patients who underwent TV surgery during follow-up were younger and more likely to have acute severe TR, while other clinical and echocardiographic characteristics did not significantly differ between groups.

Patients who underwent TV surgery during a 4-year follow-up had lower 4-year mortality compared to the conservatively managed patients (24 vs. 69%) (Supplementary Figure 3). The 3-year mortality of the patients undergoing either surgery or transcatheter treatment was comparable (24 vs. 35%) (Supplementary Figure 3).

## Discussion

The salient findings of this study are: (1) the high burden of co-morbidities in this patient population, in particular, the high prevalence of AFib, severe renal failure, PHT, and RV dysfunction; (2) the dismal mid-term prognosis of patients with severe TR (estimated all-cause mortality 50 and 69% at 2 and 4 years, respectively); (3) the low rate of interventions (<10% during the study period) performed in this patient population even when referred to a tertiary care center.

This analysis shows that patients diagnosed with severe TR are elderly with mostly secondary TR etiology, usually related to left-sided heart disease and associated PHT (3, 10, 13), which well aligns with previous studies (4, 14). About one-third of the patients had a CIED RV lead in place without impact on prognosis, while the causal relationship requires further attention.

Most of the patients in our study (68%) had AFib and its prevalence was comparable to the one observed in a cohort of US patients with severe TR (14). AFib can be both the cause or the consequence of TR (15), for which reason subclassification into atrial and ventricular secondary TR has been recently proposed (16). In ventricular secondary TR, the RV is particularly dilated (infra-annular) and the leaflets are strongly retracted, whilst in atrial secondary TR the annulus is dilated and the leaflets are less retracted. Annular dimensions correlate closely with the severity of secondary TR due to the absence of a fibrous skeleton (17). The specific prognosis of these different TR phenotypes requires further investigations.

A close association between TR and renal failure has been previously described (1, 18). Both low cardiac output and congestion due to increased central venous pressure in patients with clinically significant TR may lead to decreased renal perfusion and subsequent dysfunction. This may also be accentuated by RV dysfunction that was prevalent in our study (19). The concept of “renal tamponade” has been recently introduced and may apply to severe TR with or without RV dysfunction (20).

The cumulative mortality of 50 and 69% at 2- and 4-year follow-up, respectively, are similar to previous reports. Our results show similar 2-year mortality for untreated severe TR and untreated symptomatic severe aortic stenosis (21). Despite comparable prevalence and impact on clinical outcome (2), severe TR is less frequently contemplated as a cause of heart failure symptoms and rarely treated. In our cohort, only 9% of the patients underwent an intervention of any kind during follow-up. As possible explanations, late presentation with irreversible RV dysfunction leading to high surgical risk can be assumed along with limited availability and expertise in the domain of transcatheter interventions in the past years. The advancement of transcatheter tricuspid valve repair (TTVr) interventions is expected to address this unmet need. The 2021 ESC/EACTS Guidelines for the management of valvular heart disease first recommend evaluating transcatheter treatment in symptomatic, inoperable patients with secondary TR (IIb, level of evidence C) (22, 23). Dedicated expertise and an interdisciplinary Heart Team are essential to select patients and implement these novel techniques into clinical practice (16).

### Predictors of 2-year mortality

Besides age, and reduced LVEF, our analysis identified severe renal failure as a strong predictor of mortality. This finding is consistent with a previous study (24), and may reflect both the burden of co-morbidities leading to renal failure, as well as the end-organ damage that accompanies severe TR due to venous congestion and low cardiac output.

Patients with a large coaptation gap leading to “massive” (vena contracta width ≥14 mm) or “torrential” TR (vena contracta width ≥21 mm) had higher mortality in our cohort. Similarly, two other recent studies identified the same threshold as a predictor of cardiovascular death, admission for heart failure, and poor hemodynamics (25, 26). These findings support the use of the proposed new multi-parametric scheme for TR grading (7, 27) that has been adopted in several interventional studies (28, 29). This underlines the incremental prognostic value of this extended grading scheme in untreated patients.

### The role of CIED RV lead

Almost one-third of the patients in our cohort had a CIED lead crossing the TV. When misplaced, CIED RV leads can interact with the leaflets and cause TR (30, 31). About 10–33% of patients develop or worsen TR after implantation of a CIED RV lead (32–34), while leadless pacemaker may also interact with valve function (35), particularly when deployed at the septum close to the TV annulus (36). This has clinical implications since it has been shown that increased TR following RV pacing correlates with the subsequent risk of hospitalization for heart failure (33). However, limited awareness and the lack of prospective evidence still limit precise disease characterization. A recent study showed that TEE-guided pacemaker and ICD implantation was able to prevent TR worsening compared to standard lead implantation guided by fluoroscopy (37). Close echocardiographic follow-up may play a role in the early detection of CIED RV lead-related TR.

### Study limitations

This study has inherent limitations due to its retrospective and observational nature. Further, it includes only patients referred to a tertiary care center, so that a selection bias is likely. Although its relevance in TR patients is not well known, information about medical treatment has not been systematically collected in this study. Our work does not allow broad epidemiological conclusions, since only patients referred for echocardiography have been included. The low number of patients treated limited comparisons between groups. Finally, the maximal duration of follow-up was limited to 4 years.

## Conclusion

Our study shows the high burden of morbidity and the dismal survival of patients with severe TR. It also highlights the therapeutic need, since the vast majority of patients were left untreated. Additionally, CIED RV lead-associated TR was prevalent suggesting a need for more attention in clinical routine and research.

## Data availability statement

The data analyzed in this study is subject to the following licenses/restrictions: Pseudonymized dataset. Requests to access these datasets should be directed to MW.

## Ethics statement

The studies involving human participants were reviewed and approved by Bern Ethics Committee. The patients/participants provided their written informed consent to participate in this study.

## Author contributions

MW, DS, FP, and BC collected data, made most of the statistical analyses, and wrote most of the manuscript. NB, AR, JB, DR, TP, PW, and SW revised the manuscript for important intellectual content. MW had full access to the data and is the guarantor of the study. All authors contributed to the article and approved the submitted version.
