# Supplementary figures and images for: Natural history and mid-term prognosis of severe tricuspid regurgitation: A cohort study
Source: Front Cardiovasc Med. 2023 Jan 9;9:1026230. doi: 10.3389/fcvm.2022.1026230 (PMC9870052; doi:10.3389/fcvm.2022.1026230)

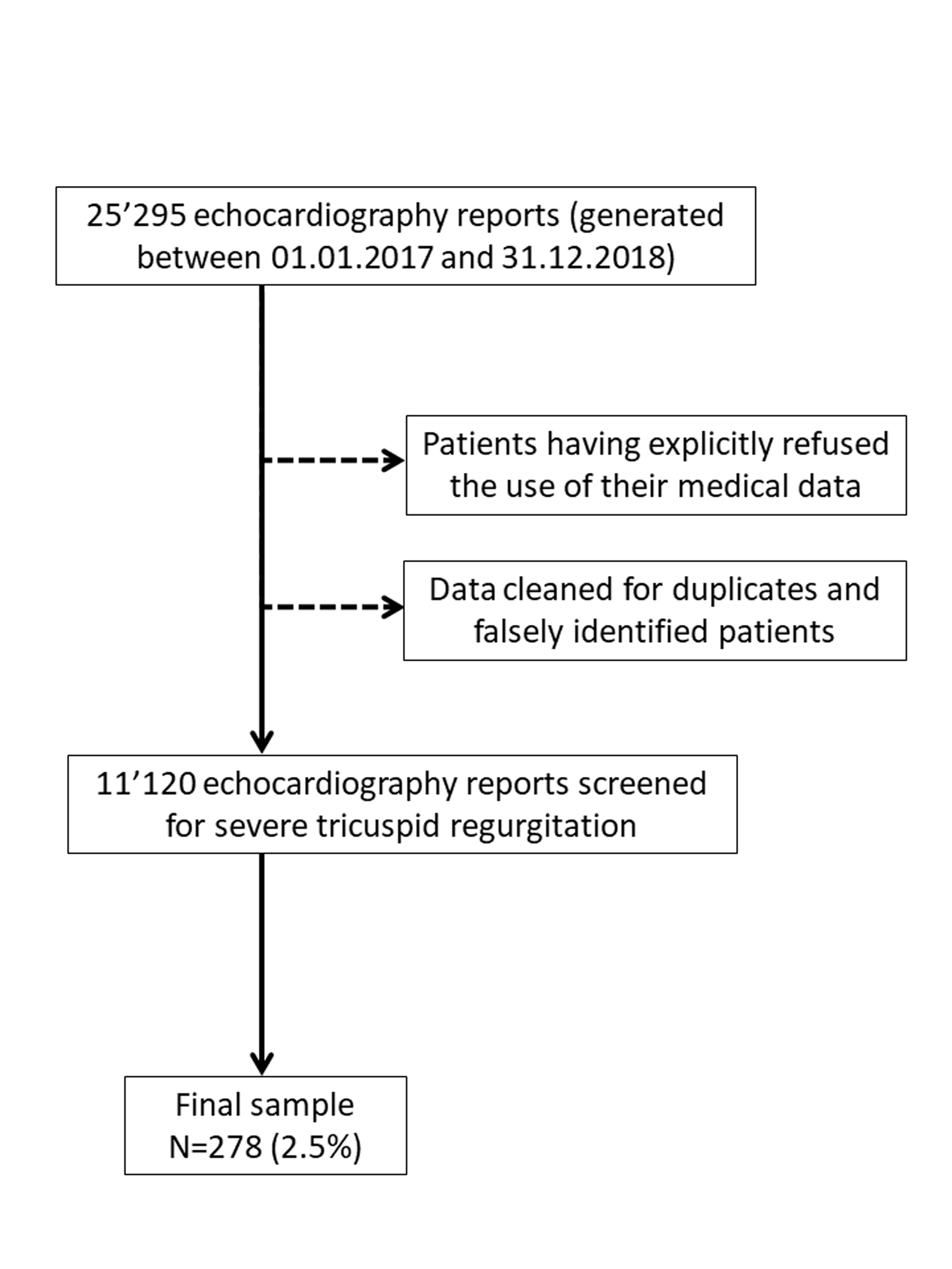

Supplement: Supplementary file 1 [file Image_1.TIF]

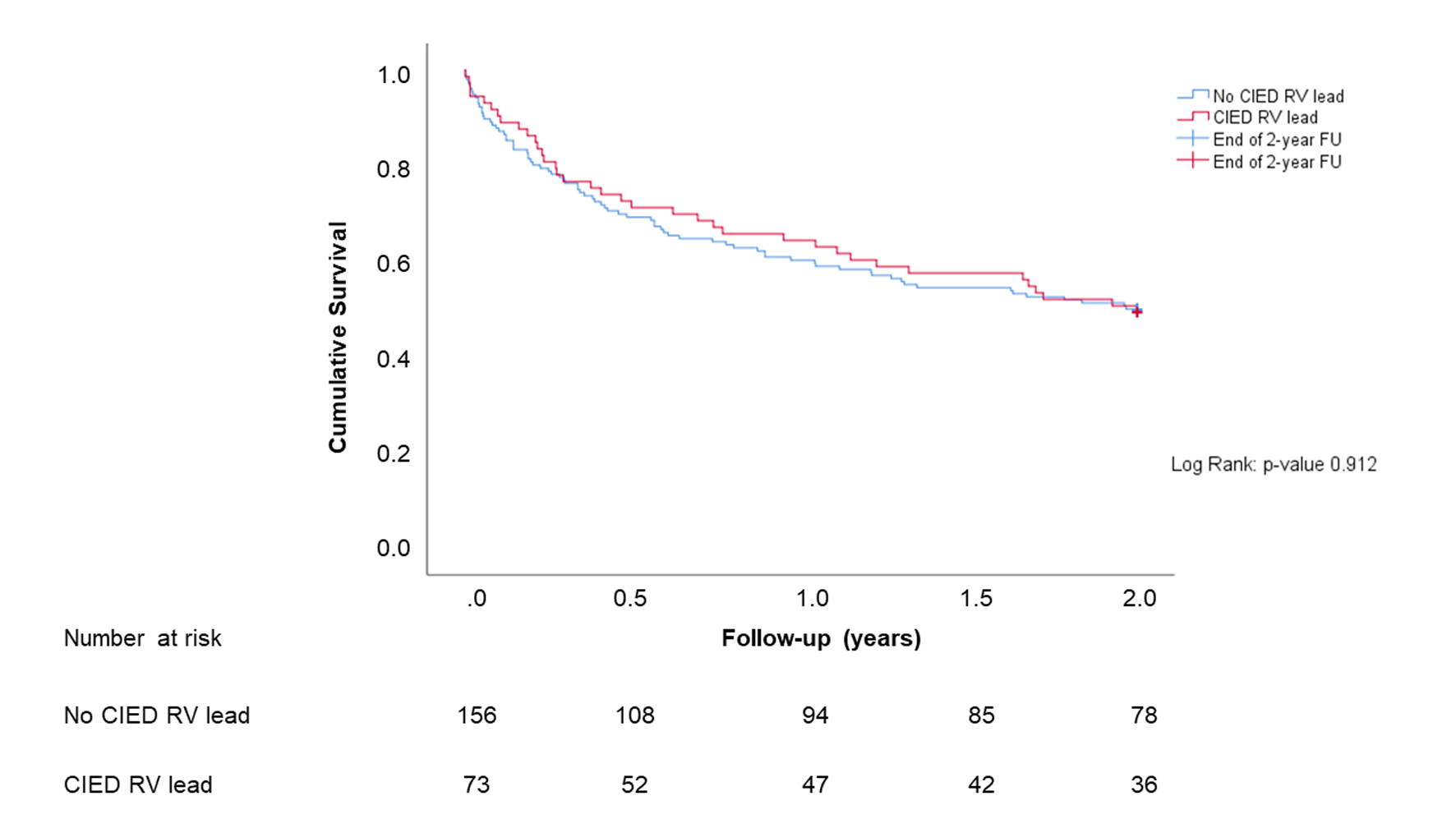

Supplement: Supplementary file 2 [file Image_2.TIF]

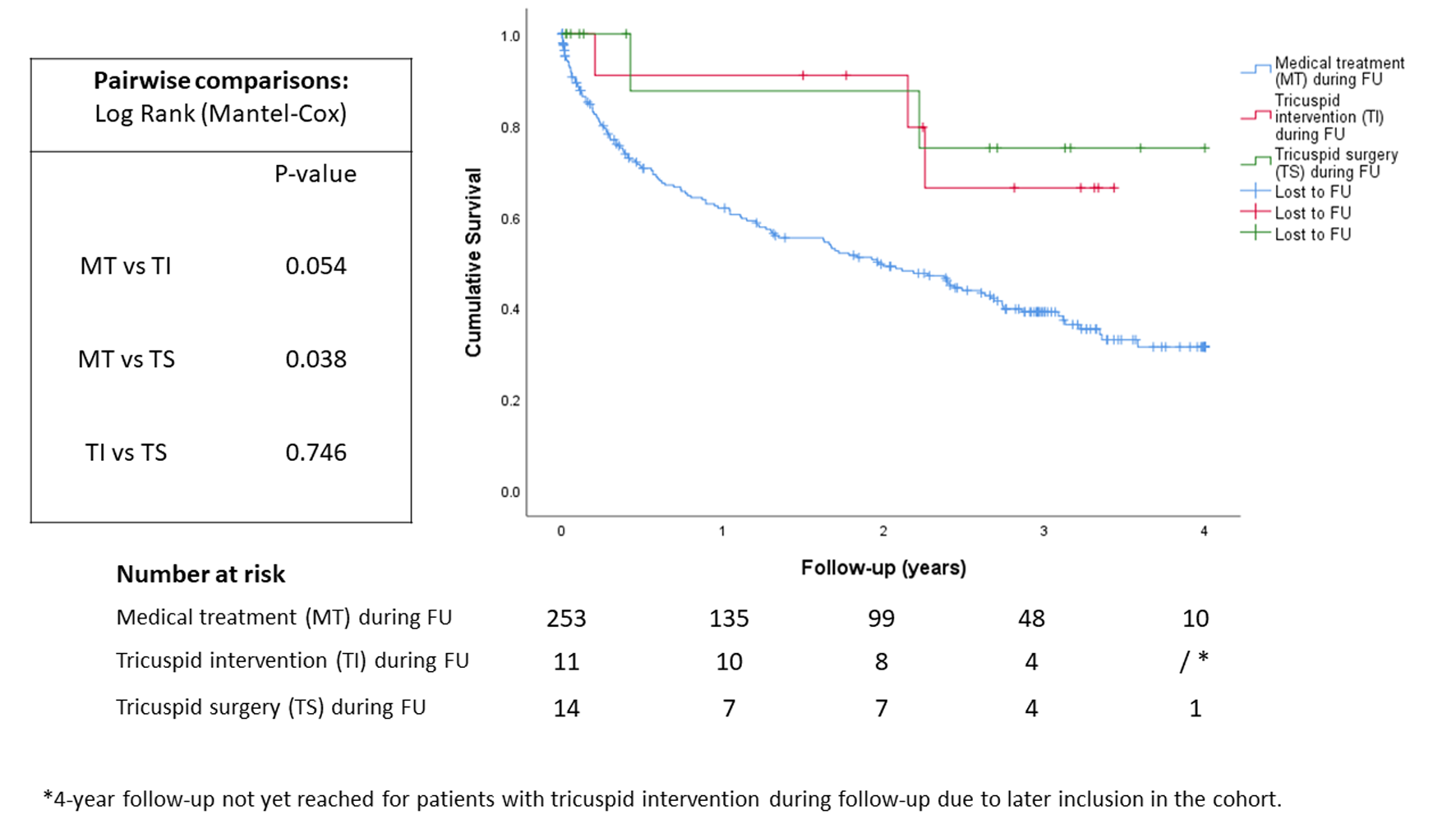

Supplement: Supplementary file 3 [file Image_3.TIF]
