# Supplementary material for: Natural history and mid-term prognosis of severe tricuspid regurgitation: A cohort study
Source: Front Cardiovasc Med. 2023 Jan 9;9:1026230. doi: 10.3389/fcvm.2022.1026230 (PMC9870052; doi:10.3389/fcvm.2022.1026230)
Supplement: Supplementary file 4 [file Data_Sheet_1.docx]

**Supplementary Materials**

**Supplementary** **Figure 1**: Number of patients retained for analysis (flowchart)

**Supplementary** **Figure 2**: Estimated cumulative survival (Kaplan-Meier curves) of patients with and without cardiac implantable electronic device (CIED) RV lead up to 2-year follow-up

**Supplementary** **Figure 3**: Estimated cumulative survival (Kaplan-Meier curves) of patients according to their treatment strategy during 4-year follow-up

**Supplementary Table 1**. Previous open-heart surgery

| Total number of previous open-heart surgeries | 111 |
| --- | --- |
| Aortic valve replacement | 35 (31.5) |
| Mitral valve replacement | 10 (9.0) |
| Mitral valve repair | 5 (4.5) |
| Tricuspid valve repair | 3 (2.7) |
| Coronary artery bypass grafting (CABG) | 40 (36.0) |
| OHT/Ventricular assistance device | 13 (11.7) |
| Ventricle septum defect closure | 3 (2.7) |
| Atrial septum defect closure | 1 (0.9) |
| Correction of transposition of the great arteries | 1 (0.9) |

Results are expressed as absolute number (percentage). OHT: Orthotopic heart transplantation

**Supplementary Table 2**. Interventions during 4-year follow-up

| N | 278 |
| --- | --- |
| TV intervention | 11 (4.0) |
| Time from diagnosis to intervention, days, median [IQR] | 196 [41-657] |
| TV surgery | 14 (5.0) |
| Time from diagnosis to surgery, days, median [IQR] | 55 [5-246] |
| Type of TV intervention |  |
| Tricuspid TEER | 5 (1.8) |
| Valve replacement (EVOQUE) | 1 (0.4) |
| Direct annuloplasty | 5 (1.8) |
| Bicaval stenting | 2 (0.7) |
| Types of tricuspid valve surgery |  |
| Repair | 13 (4.3) |
| Replacement | 1 (0.4) |
| Others |  |
| Mitral valve repair | 2 (0.7) |
| Mitral valve replacement | 3 (1.0) |
| Aortic valve replacement | 5 (1.8) |
| Coronary artery bypass graft | 1 (0.4) |
| PFO closure | 1 (0.4) |
| LAA closure | 2 (0.7) |

Results are expressed as number of patients (percentage) or median days [IQR]. TEER: Transcatheter edge-to-edge repair; PFO: patent foramen ovale; LAA: Left atrial appendage. NB: The number of tricuspid interventions (13) exceeds the number of patients who had a tricuspid intervention (11) because two patients had a tricuspid direct annuloplasty combined with TEER during follow-up.
